# Concurrent *Plasmodium vivax* Malaria and Dengue

**DOI:** 10.3201/eid1211.060341

**Published:** 2006-11

**Authors:** Stan Deresinski

**Affiliations:** * Stanford University, Stanford, California, USA;; † Santa Clara Valley Medical Center, San Jose, California, USA

**Keywords:** malaria, Plasmodium vivax, dengue, letter

To the Editor: The first report of a patient with concurrent malaria (Plasmodium falciparum) and dengue was recently published in this journal (*1*). Herein is presumably the first report of concurrent dengue and malaria due to P. vivax.

A 27-year-old woman experienced the onset of myalgia on December 11, 2003, 1 day before returning home to California from India after a 3-month sojourn in that country. The following day she had chills and a low-grade fever, and she visited an urgent care center. A presumptive diagnosis of influenza was made, and she was discharged with antipyretic therapy. A single malaria smear was subsequently reported to be negative for Plasmodium.

On December 15, she sought treatment at a hospital emergency department at 3:30 A.M.. with an oral temperature of 39.5°C. Her leukocyte count was 4,300 × 10^9^/L hemoglobin level 119 g/L, and platelet count 157,000 × 10^9^/L. A diagnosis of probable viral syndrome was made, and she was discharged with antipyretic therapy. She returned to the urgent care center the following day with a temperature of 38.6°C, and a 10-day course of amoxicillin was prescribed on discharge.

On December 18, she sought treatment from an infectious disease specialist. She had an oral temperature of 39.3°C and was dehydrated, which led to her admission to the hospital. Results of the examination were otherwise unremarkable.

She reported that she had lived in the United States for the last 4 years, after moving there from India. During her recent trip to India, she had spent most of the time in Surat, followed by 3 days in Mumbai. She indicated that she had had malaria several times while living in India. She received no vaccinations before her trip and took no malaria prophylaxis; she believed she was likely immune and, in addition, she was concerned about taking medications while breastfeeding her 6-month-old child. The child received no prophylaxis or other medical preparation for the trip but remained well.

Her leukocyte count was 4,500 times; 10^9^/L with 50% polymorphonuclear leukocytes, 18% band forms, 3% myelocytes, and 1% metamyelocytes. Hemoglobin level was 11.1 g/L, and platelet count was now 98.0 × 10^9^/L. P. vivax was seen on blood smear, and the patient was treated with chloroquine with rapid resolution of her fever, followed by administration of primaquine, during which course she avoided breastfeeding. In addition, enzyme immunoassays for dengue virus were performed on December 19 (immunoglobulin G [IgG] 6.55; IgM 4.17) and subsequently repeated on December 31 (IgG 7.29; IgM 1.07), indicating an acute infection. Viral isolation was not attempted.

I agree with Charrel and colleagues (*1*) that, although only 2 cases have now been reported, concurrent dengue and malaria is probably not a rare event. This conclusion is supported by a recent report from Pakistan (*2*).
